# Metabolic dysfunction-associated fatty liver disease increases the risk of complications after radical resection in patients with hepatocellular carcinoma

**DOI:** 10.1186/s12957-024-03385-7

**Published:** 2024-05-03

**Authors:** Ke-Gong Xiong, Kun-Yu Ke, Jin-Feng Kong, Tai-Shun Lin, Qing-Biao Lin, Su Lin, Yue-Yong Zhu

**Affiliations:** 1 Department of Hepatology, Mengchao Hepatobiliary Hospital of Fujian Medical University, Fuzhou, 350001 China; 2 Department of Hepatology, Hepatology Research Institute, the First Affiliated Hospital of Fujian Medical University, Fuzhou, 350001 China; 3 Fujian Clinical Research Center for Liver and Intestinal Diseases, Fuzhou, 350001 China

**Keywords:** Metabolic dysfunction-associated fatty liver disease, Hepatocellular carcinoma, Radical resection, Complications

## Abstract

**Background and aims:**

The prevalence of metabolic dysfunction-associated fatty liver disease (MAFLD) in hepatocellular carcinoma (HCC) patients is increasing, yet its association with postoperative complications of HCC remains unclear. The aim of this study was to investigate the impact of MAFLD on complications after radical resection in HCC patients.

**Methods:**

Patients with HCC who underwent radical resection were included. Patients were stratified into MAFLD group and non-MAFLD group. Clinical features and post-hepatectomy complications were compared between the two groups, and logistic regression analysis was used to determine independent risk factors associated with post-hepatectomy complications.

**Results:**

Among the 936 eligible patients with HCC who underwent radical resection, concurrent MAFLD was diagnosed in 201 (21.5%) patients. Compared to the non-MAFLD group, the MAFLD group exhibited a higher incidence of complications, including infectious and major complications after radical resection in HCC patients. The logistic regression analysis found that MAFLD was an independent risk factor for complications, including infectious and major complications in HCC patients following radical resection (OR 1.565, 95%CI 1.109–2.343, *P* = 0.012; OR 2.092, 95%CI 1.386–3.156, *P* < 0.001; OR 1.859, 95% CI 1.106–3.124, *P* = 0.019; respectively). Subgroup analysis of HBV-related HCC patients yielded similar findings, and MAFLD patients with type 2 diabetes mellitus (T2DM) exhibited a higher incidence of postoperative complications compared to those without T2DM (all *P* < 0.05).

**Conclusions:**

Concurrent MAFLD was associated with an increased incidence of complications after radical resection in patients with HCC, especially MAFLD with T2DM.

## Introduction

The prevalence of nonalcoholic fatty liver disease (NAFLD) has progressively increased over the past few decades, reaching a level almost equivalent to that of obesity and has emerged as the foremost chronic liver disease in contemporary times, posing a threat to 25% of global human health [1]. With the deepening understanding of the etiology and pathogenesis of NAFLD, it was revised to metabolic dysfunction-associated fatty liver disease (MAFLD) by an international panel of experts from 22 countries in 2020. The diagnosis of MAFLD is etiologically oriented and recognizes the coexistence of MAFLD with other liver diseases, thereby providing a more comprehensive understanding of its pathogenesis and facilitating patient classification and management [2]. Compared to NAFLD prevalence, MAFLD prevalence was higher, posing an elevated risk of overall mortality [3].

Primary liver cancer (PLC) is ranked sixth in incidence and third in mortality among 36 types of cancers across 185 countries worldwide [4]. It is estimated that there were approximately 906,000 new patients and nearly 830,000 deaths from PLC globally in 2020. Hepatocellular carcinoma (HCC) is the most prevalent histological subtype of PLC, accounting for approximately 80%-90% [4]. The HBV infection is the predominant risk factor for HCC in China, accounting for about 90% [5, 6]. Currently, hepatectomy remains the most efficacious treatment option for early-stage HCC [7, 8]. However, the incidence of postoperative complications remains high, particularly in relation to ascites, infectious and major complications, exerting detrimental effects on patient prognosis [9–12].

The prevalence of MAFLD in the global population is gradually increasing, leading to an increased number of HCC patients being diagnosed with MAFLD. An Italian Liver Cancer Center study showed that out of 6882 patients diagnosed with HCC, 4706 (68.4%) patients were found to have MAFLD [13]. A Chinese study showed that among 514 HBV-HCC patients who underwent radical resection, MAFLD was detected in 117 (22.8%) patients [14]. MAFLD serves as a significant risk factor for the development of HCC and warrants careful consideration from clinicians regarding its potential impact on post-hepatectomy complications. However, the relationship between MAFLD and post-hepatectomy complications in patients with HCC remains unclear. The aim of this study was to evaluate the predictive value of the MAFLD on complications after radical resection in HCC patients.

## Methods

### Study population

All HCC patients who were underwent radical resection at Mengchao Hepatobiliary Hospital of Fujian Medical University from January 2015 to December 2020 were retrospectively collected. The inclusion criteria were patients with HCC: confirmed through pathological examination following the initial radical resection, favorable liver function reserve (Child–Pugh grade A or B). The exclusion criteria were as follows: hepatocellular-cholangiocarcinoma (HCC—ICC); accompanied by other malignant tumors; invasive treatment before operation [transcatheter hepatic arterial chemoembolization (TACE) or radiofrequency ablation (RFA)]; multiple intrahepatic metastases, adjacent organ invasion or distant metastases; incomplete clinical data.

### Data collection

The clinical data were retrospectively extracted from medical records, including baseline data [(age, sex, height, weight, alcohol consumed, hypertension, type 2 diabetes mellitus (T2DM), albumin (ALB), total bilirubin (TBIL), alanine aminotransferase (ALT), aspartate aminotransferase (AST), total cholesterol (TC), triglyceride (TG), low-density lipoprotein cholesterol (LDL-C), high-density lipoprotein cholesterol (HDL-C), fasting plasma glucose (FPG), HbA1c, high-sensitive C-reactive protein (hs-CRP), prothrombin time (PT), white blood cell (WBC), hemoglobin (HB), platelet (PLT), alpha-fetoprotein (AFP), HBsAg, HBV DNA, Child–Pugh grading, BCLC staging, etc.], surgical and tumor data [surgical methods (open surgery or laparoscopic surgery), intraoperative bleeding, intraoperative blood transfusion, pathological types of tumor tissue, tumor size, tumor number, tumor cell differentiation, tumor capsule, microvascular invasion (MVI), microsatellite lesions, etc.] and postoperative complications (pleural fluid, ascites, abdominal hemorrhage, infection, liver failure, bile leakage, hepatic encephalopathy, cardiovascular events and death within 30 days, etc.).

### Definition

The diagnosis of MAFLD was confirmed by hepatic histology, which revealed the presence of hepatic steatosis and met one of the following criteria: BMI ≥ 23 kg/m^2^, T2DM, or metabolic dysregulation (MD) [2]. Lean MAFLD referred to patients with a BMI < 23 kg/m^2^ who also met the diagnostic criteria for MAFLD [15–17]. The criteria for radical resection of HCC were as follows: the liver resection margin should be ≥ 1 cm from the tumor boundary; in patients where the resection margin was less than 1 cm, histological examination of the liver resection section should reveal no residual tumor cells [18]. Excessive alcohol consumption: alcohol intake ≥ 30 g/day for men and ≥ 20 g/day for women [19]. Postoperative complications were defined as conditions that cause discomfort or abnormal auxiliary examination results secondary to radical resection. The severity of postoperative complications was evaluated using the comprehensive complication index (CCI) [20]. The presence of CCI ≥ 26.2 indicates major complications while CCI < 26.2 suggests general complication [21, 22].

### Statistical analysis

SPSS 22.0 was utilized for conducting statistical analysis. Continuous variables were described using the median (interquartile range, IQR), while inter-group comparisons were performed using either T-test or Mann–Whitney U test. Categorical variables were presented as frequency with corresponding percentages (%), and inter-group comparisons were conducted using either a χ^2^ test or Fisher exact test. Univariate and multivariate logistic regression analyses were conducted to examine the risk factors associated with complications after radical resection in HCC patients. Variables with *P* < 0.05 in the univariate analysis were considered as candidate variables for inclusion in the logistic multivariate analysis. The odds ratio (OR) and its corresponding 95% CI were calculated. The forest plot illustrating the influencing factors of complications after radical resection in HCC patients was generated using software GraphPad Prism 8. *P* values < 0.05 indicated statistical significance.

## Results

### Baseline characteristics of HCC patients

**Fig. 1:**
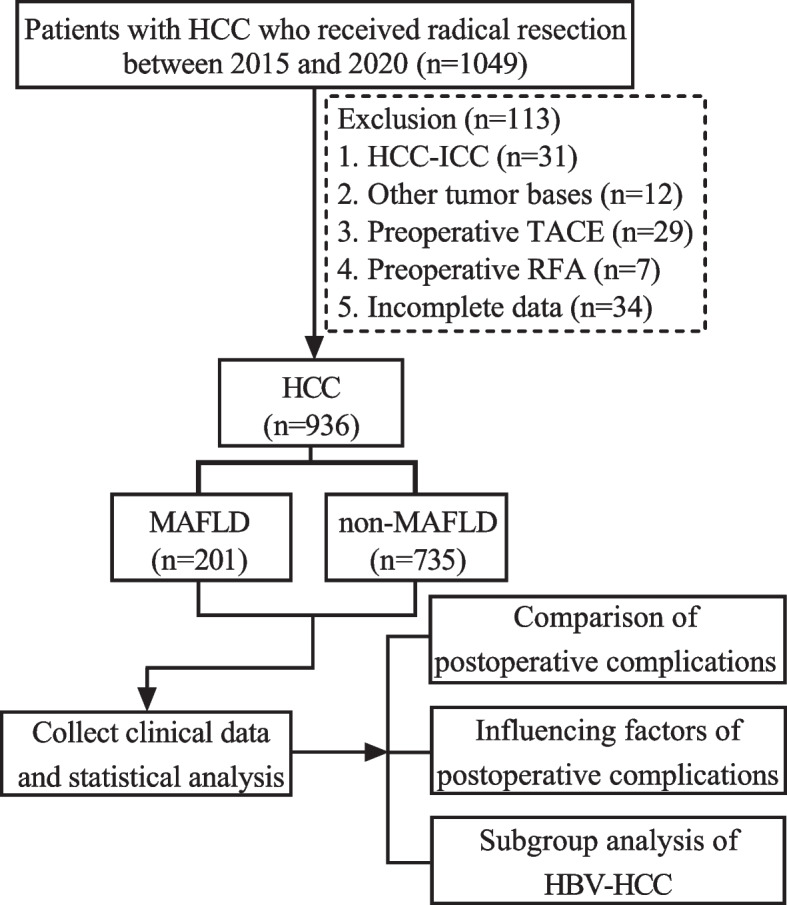
Flow chart for the selection of the study population

The study cohort was selected and depicted in Fig. 1. A total of 936 HCC patients who underwent radical resection were enrolled in this study, comprising of 764 (81.6%) males and 172 (18.4%) females. Among the study population, the median age of patients was 57 (48.0–64.0) years, BMI ≥ 23 kg/m^2^ was observed in 477 (51.0%) patients. The prevalence of T2DM, MD, excessive alcohol consumption and HBsAg-positivity were 140 (15.0%), 324 (34.6%), 103 (11.0%), and 853 (91.1%) patients, respectively. Additionally, the median tumor diameter measured 4.0 (2.7–6.4) cm. The majority of these tumors were solitary, accounting for 87.4% (818/936) (Table 1).

**Table 1:** Baseline characteristics of HCC patients

Variables	Patients (*n* = 936)	MAFLD (*n* = 201)	non-MAFLD (*n* = 735)	*P* value
Age (years)	57.0 (48.0–64.0)	57.0 (48.0–62.0)	57.0 (49.0–64.0)	0.355
Male	764 (81.6%)	159 (79.1%)	605 (82.3%)	0.298
BMI (kg/m^2^)	22.9 (21.0–25.0)	24.2 (22.6–25.8)	22.3 (20.7–24.5)	< 0.001
BMI ≥ 23 (kg/m^2^)	477 (51.0%)	158 (78.6%)	319 (43.4%)	< 0.001
T2DM	140 (15.0%)	56 (27.9%)	84 (11.4%)	< 0.001
MD	324 (34.7%)	101 (50.2%)	223 (30.4%)	< 0.001
Excessive alcohol consumed	103 (11.0%)	27 (13.4%)	76 (10.3%)	0.214
HBsAg-positive	853 (91.1%)	178 (88.6%)	675 (91.8%)	0.147
HBV DNA (≥ 500 IU/mL)	763 (81.5%)	165 (82.1%)	598 (81.4%)	0.813
Cirrhosis	765 (81.7%)	171 (85.1%)	594 (80.8%)	0.166
Child–Pugh grade				0.646
A	864 (92.3%)	184 (91.5%)	680 (92.5%)	
B	72 (7.7%)	17 (8.5%)	55 (7.5%)	
Leukocyte count (× 10^9^/L)	5.5 (4.6–6.6)	5.5 (4.6–6.6)	5.5 (4.5–6.6)	0.523
Hemoglobin (g/L)	143.0 (138.0–152.3)	142.0 (136.0–153.5)	143.0 (139.0–152.0)	0.743
Platelet count (× 10^9^/L)	168.0 (146.2–207.0)	171.0 (148.0–207.5)	166.0 (146.0–207.0)	0.321
Prothrombin time (s)	13.3 (12.7–13.8)	13.3 (12.6–13.7)	13.3 (12.7–13.9)	0.082
Albumin (g/L)	40.0 (38.0–43.0)	40.0 (38.0–44.0)	40.0 (38.0–43.0)	0.513
Total bilirubin (µmol/L)	16.3 (12.0–21.8)	16.6 (11.7–22.8)	16.3 (12.0–21.6)	0.834
ALT (IU/L)	33.0 (23.0–49.0)	36.0 (27.0–51.0)	32.0 (23.0–48.0)	0.012
AFP (µg/L)	48.0 (6.2–611.6)	39.7 (5.4–215.1)	48.0 (6.3–777.1)	0.102
Tumor diameter (cm)	4.0 (2.7–6.4)	4.0 (3.0–5.9)	4.0 (2.5–6.5)	0.643
Number of tumors				0.524
1	818 (87.4%)	173 (86.1%)	645 (87.8%)	
≥ 2	118 (12.6%)	28 (13.9%)	90 (12.2%)	
Tumor cell differentiation				0.062
Well	12 (1.3%)	4 (2.0%)	8 (1.1%)	
Moderate	428 (45.7%)	78 (38.8%)	350 (47.6%)	
Poor	496 (53.0%)	119 (59.2%)	377 (51.3%)	
Tumor capsule				0.181
Complete	208 (22.2%)	48 (23.9%)	160 (21.8%)	
Incomplete	588 (62.8%)	116 (57.7%)	472 (64.2%)	
No tumor capsule	140 (15.0%)	37 (18.4%)	103 (14.0%)	
Microvascular invasion	518 (55.3%)	109 (54.2%)	409 (55.6%)	0.720
Microsatellite lesions	199 (21.3%)	37 (18.4%)	162 (22.0%)	0.265
BCLC stage				0.876
0	8 (0.9%)	2 (1.0%)	6 (0.8%)	
A	841 (89.9%)	182 (90.5%)	659 (89.7%)	
B	87 (9.3%)	17 (8.5%)	70 (9.5%)	
Surgical method				0.847
Open	462 (49.4%)	98 (48.8%)	364 (49.5%)	
Laparoscopic	474 (50.6%)	103 (51.2%)	371 (50.5%)	

*HCC* hepatocellular carcinoma, *MAFLD* metabolic dysfunction-associated fatty liver disease, *BMI* body mass index, *T2DM* type 2 diabetes mellitus, *MD* metabolic dysregulation, *ALT* alanine aminotransferase, *AFP* alpha-fetoprotein, *BCLC* Barcelona Clinic Liver Cancer

The HCC patients were classified into the MAFLD group (201, 21.5%) and the non-MAFLD group (735, 78.5%) based on the presence or absence of MAFLD. In comparison to the non-MAFLD group, the MAFLD group exhibited a higher median BMI (24.2 vs 22.3 kg/m^2^, *P* < 0.001) and a greater proportion of patients with combined BMI ≥ 23 kg/m^2^ (78.6% vs 43.4%, *P* < 0.001). Moreover, the prevalence rates of T2DM and MD in the MAFLD group were significantly higher compared to the non-MAFLD group (27.9% vs 11.4%, *P* < 0.001; 50.2% vs 30.4%, *P* < 0.001; respectively). Additionally, the ALT levels were also significantly higher in the MAFLD group compared to the non-MAFLD group (36.0 vs 32 IU/L, *P* = 0.012). No significant differences were observed between both groups in terms of other characteristics (all *P* > 0.05) (Table 1).

### Complications after radical resection in HCC patients

The overall morbidity rate of complications after radical resection in HCC patients was 21.0% (197/936). Classification of postoperative complications according to CCI: CCI (8.7–20.8) 49 (5.2%) patients, CCI (20.9–26.1) 67 (7.2%) patients, CCI (26.2–33.6) 26 (2.7%) patients, CCI (33.7–42.3) 18 (1.9%) patients, CCI (42.4–46.1) 16 (1.7%) patients, and CCI (46.2–100) 22 (2.4%) patients. Additionally, major complications (CCI ≥ 26.2) occurred in 80 (8.5%) patients (Tables 2 and 3).

**Table 2:** Incidence of postoperative complications in HCC patients

Complications	n (%)
Ascites	108 (11.5%)
Pleural effusion	105 (11.2%)
Intra-abdominal infection	78 (8.3%)
Pneumonia	72 (7.7%)
Liver failure	26 (2.8%)
Wound infection	18 (1.9%)
Intra-abdominal hemorrhage	14 (1.5%)
Hepatic encephalopathy	10 (1.1%)
Bile leakage	5 (0.5%)
Sepsis	4 (0.4%)
Acute renal failure	1 (0.1%)
Cardiovascular event	1 (0.1%)
Death	10 (1.1%)

*HCC* hepatocellular carcinoma

**Table 3:** CCI classification of postoperative complications in HCC patients

CCI	n (%)
0	739 (79.0%)
8.7–20.8	49 (5.2%)
20.9–26.1	67 (7.2%)
26.2–33.6	26 (2.7%)
33.7–42.3	18 (1.9%)
42.4–46.1	16 (1.7%)
46.2–100	22 (2.4%)
≥ 26.2	80 (8.5%)

*HCC* hepatocellular carcinoma, *CCI* comprehensive complication index

The overall incidence of postoperative complications in the MAFLD group was higher compared to the non-MAFLD group (27.4% vs 19.3%, *P* = 0.013). Moreover, the MAFLD group exhibited a higher occurrence of postoperative infectious and major complications (CCI ≥ 26.2) compared to the non-MAFLD group (23.4% vs 13.5%, *P* = 0.001; 12.4% vs 7.5%, *P* = 0.026, respectively). Further analysis found that the MAFLD group exhibited a higher incidence of postoperative complications, including pleural effusion, intra-abdominal infection, liver failure, wound infection, and death within 30 days (all *P* < 0.05). However, there were no statistically significant differences observed in other complications between the two groups (Table 4).

**Table 4:** Comparison of complications between MAFLD group and non-MAFLD group

Variables	MAFLD (*n* = 201)	non-MAFLD (*n* = 735)	*P* value
Complications	55 (27.4%)	142 (19.3%)	0.013
Infectious complications	47 (23.4%)	99 (13.5%)	0.001
Major complications (CCI ≥ 26.2)	25 (12.4%)	55 (7.5%)	0.026
Pleural effusion	33 (16.4%)	75 (10.2%)	0.015
Ascites	29 (14.4%)	76 (10.3%)	0.104
Intra-abdominal infection	26 (12.9%)	52 (7.1%)	0.008
Pneumonia	21 (10.4%)	51 (6.9%)	0.098
Liver failure	10 (5.0%)	16 (2.2%)	0.032
Wound infection	8 (4.0%)	10 (1.4%)	0.017
Intra-abdominal hemorrhage	5 (2.5%)	9 (1.2%)	0.191
Hepatic encephalopathy	2 (1.0%)	8 (1.1%)	0.909
Bile leakage	2 (1.0%)	3 (0.4%)	0.312
Sepsis	2 (1.0%)	2 (0.3%)	0.164
Acute renal failure	0 (0)	2 (0.3%)	0.459
Cardiovascular event	1 (0.5%)	0 (0)	0.056
Death	6 (3.0%)	4 (0.5%)	0.009

*MAFLD* metabolic dysfunction-associated fatty liver disease, *CCI* comprehensive complication index

### Influencing factors of complications after radical resection in HCC patients

Univariate logistic regression analysis found that MAFLD was identified as a significant risk factor of complications after radical resection in HCC patients (OR 1.573, 95%CI 1.097–2.255, *P* = 0.014). Additionally, age ≥ 60 years, male, T2MD, tumor diameter ≥ 5 cm, number of tumors ≥ 2, MVI, Child–Pugh grade B and open surgery were significantly associated with post-hepatectomy complications in HCC patients (all *P* < 0.05) (Table 5).

**Table 5:** Univariate and multivariate analysis of complications after radical resection in HCC patients

Variables	Univariate		Multivariate	
	OR (95% CI)	*P* value	OR (95% CI)	*P* value
MAFLD	1.573 (1.097–2.255)	0.014	1.565 (1.109–2.343)	0.012
Age ≥ 60 years	1.666 (1.215–2.286)	0.002	1.820 (1.306–2.534)	< 0.001
Male	0.576 (0.395–0.838)	0.004	0.729 (0.487–1.092)	0.126
BMI ≥ 23 kg/m^2^	0.893 (0.652–1.223)	0.481		
T2DM	2.090 (1.409–3.102)	< 0.001	NA^a^	
MD	1.186 (0.856–1.645)	0.305		
Alcohol consumed	1.232 (0.762–1.992)	0.395		
HBV DNA ≥ 500 IU/mL	1.336 (0.869–2.052)	0.186		
AFP ≥ 400 µg/L	0.914 (0.645–1.295)	0.612		
Cirrhosis	1.091 (0.722–1.650)	0.680		
Maximum tumor diameter ≥ 5 cm	1.719 (1.252–2.361)	0.001	1.291 (0.915–1.820)	0.146
Tumor number ≥ 2	1.708 (1.110–2.627)	0.015	1.594 (1.017–2.499)	0.042
Tumor cell differentiation (well or moderate vs. poor)	1.191 (0.867–1.635)	0.281		
Tumor capsule (complete or incomplete vs. no)	0.944 (0.619–1.441)	0.790		
Microvascular invasion	1.857 (1.335–2.584)	< 0.001	1.731 (1.219–2.458)	0.002
Microsatellite lesions	1.167 (0.802–1.698)	0.420		
BCLC stage B	1.217 (0.725–2.043)	0.458		
Child–Pugh grade B	2.617 (1.583–4.327)	0.016	2.433 (1.416–4.181)	0.001
Open surgery	1.809 (1.313–2.493)	< 0.001	1.511 (1.067–2.139)	0.020

*HCC* hepatocellular carcinoma, *MAFLD* metabolic dysfunction-associated fatty liver disease, *BMI* body mass index, *T2DM* type 2 diabetes mellitus, *MD* metabolic dysregulation, *AFP* alpha-fetoprotein, *BCLC* Barcelona Clinic Liver Cancer

^a^The diagnostic criteria for MAFLD include T2DM

Multivariate logistic regression analysis revealed that MAFLD was an independent risk factor of complications after radical resection in HCC patients (OR 1.565, 95%CI 1.109–2.343, *P* = 0.012). Additionally, age ≥ 60 years, number of tumors ≥ 2, MVI, Child–Pugh grade B and open surgery were also identified as significant independent risk factors of post-hepatectomy complications (all *P* < 0.05) (Table 5).

### Influencing factors of infectious complications after radical resection in HCC patients

Univariate logistic regression analysis found that MAFLD was identified as a risk factor of complications after radical resection in HCC patients (OR 1.961, 95%CI 1.328–2.894, *P* = 0.001). Additionally, age ≥ 60 years, T2MD, HBV DNA ≥ 500 IU/mL, tumor diameter ≥ 5 cm, tumor number ≥ 2, MVI, Child–Pugh grade B and open surgery were also found to be associated with an increased risk of infectious complications after radical resection in HCC patients (all *P* < 0.05) (Table 6).

**Table 6:** Univariate and multivariate analysis of infectious complications after radical resection in HCC patients

Variables	Univariate		Multivariate	
	OR (95% CI)	*P* value	OR (95% CI)	*P* value
MAFLD	1.961 (1.328–2.894)	0.001	2.092 (1.386–3.156)	< 0.001
Age ≥ 60 years	1.907 (1.336–2.722)	< 0.001	2.118 (1.453–3.088)	< 0.001
Male	0.725 (0.472–1.113)	0.141		
BMI ≥ 23 kg/m^2^	0.895 (0.629–1.275)	0.540		
T2DM	2.604 (1.711–3.962)	< 0.001	NA^a^	
MD	1.313 (0.913–1.889)	0.141		
Excessive alcohol consumed	1.352 (0.801–2.283)	0.259		
HBV DNA ≥ 500 IU/mL	2.166 (1.672–4.994)	< 0.001	2.616 (1.354–5.054)	0.004
AFP ≥ 400 µg/L	0.965 (0.654–1.425)	0.859		
Cirrhosis	1.310 (0.805–2.133)	0.277		
Maximum tumor diameter ≥ 5 cm	2.184 (1.528–3.121)	< 0.001	1.505 (1.017–2.228)	0.041
Tumor number ≥ 2	1.644 (1.021–2.647)	0.041	1.491 (0.900–2.469)	0.121
Tumor cell differentiation (well or moderate vs. poor)	1.076 (0.754–1.534)	0.687		
Tumor capsule (complete or incomplete vs. no)	0.974 (0.604–1.569)	0.913		
Microvascular invasion	1.735 (1.197–2.514)	0.004	1.582 (1.059–2.363)	0.025
Microsatellite lesions	1.150 (0.755–1.751)	0.515		
BCLC stage B	1.355 (0.773–2.378)	0.289		
Child–Pugh grade B	2.828 (1.663–4.808)	< 0.000	2.731 (1.520–4.909)	0.001
Open surgery	2.305 (1.591–3.340)	< 0.000	1.816 (1.220–2.703)	0.003

*HCC* hepatocellular carcinoma, *MAFLD* metabolic dysfunction-associated fatty liver disease, *BMI* body mass index, *T2DM* type 2 diabetes mellitus, *MD* metabolic dysregulation, *AFP* alpha-fetoprotein, *BCLC* Barcelona Clinic Liver Cancer

^a^The diagnostic criteria for MAFLD include T2DM

Multivariate logistic regression analysis showed that MAFLD was an independent risk factor of infectious complications after radical resection in HCC patients (OR 2.092, 95%CI 1.386–3.156, *P* < 0.001). The other independent risk factors included: age ≥ 60 years, HBV DNA ≥ 500 IU/mL, tumor diameter ≥ 2, MVI, Child–Pugh grade B and open surgery (all *P* < 0.05) (Table 6).

### Influencing factors of major complications after radical resection in HCC patients

Univariate logistic regression analysis found that MAFLD was a risk factor of major complications (CCI ≥ 26.2) after radical resection in HCC patients (OR 1.756, 95%CI 1.064–2.898, *P* = 0.028). Additionally, age ≥ 60 years, BMI ≥ 23 kg/m^2^, T2DM, tumor diameter ≥ 5 cm, MVI, Child–Pugh grade B and open surgery were also found to be associated with an increased risk of major complications after radical resection in HCC patients (all *P* < 0.05) (Table 7).

**Table 7:** Univariate and multivariate analysis of major complications after radical resection in HCC patients

Variables	Univariate		Multivariate	
	OR (95% CI)	*P* value	OR (95% CI)	OR (95% CI)
MAFLD	1.756 (1.064–2.898)	0.028	1.859 (1.106–3.124)	0.019
Age ≥ 60 years	1.828 (1.153–2.899)	0.010	2.038 (1.264–3.287)	0.004
Male	0.643 (0.376–1.098)	0.106		
BMI ≥ 23 kg/m^2^	0.582 (0.364–0.930)	0.024	NA^a^	
T2DM	2.372 (1.399–4.022)	0.001	NA^a^	
MD	0.845 (0.516–1.384)	0.504		
Alcohol consumed	0.759 (0.340–1.696)	0.502		
HBV DNA ≥ 500 IU/mL	1.464 (0.758–2.830)	0.257		
AFP ≥ 400 µg/L	1.033 (0.626–1.704)	0.899		
Cirrhosis	0.966 (0.537–1.738)	0.907		
Maximum tumor diameter ≥ 5 cm	1.701 (1.074–2.693)	0.024	2.665 (1.526–4.656)	0.001
Tumor number ≥ 2	1.388 (0.741–2.601)	0.307		
Tumor cell differentiation (well or moderate vs. poor)	1.310 (0.821–2.089)	0.258		
Tumor capsule (complete or incomplete vs. no)	1.569 (0.766–3.212)	0.218		
Microvascular invasion	1.803 (1.646–2.772)	< 0.001	1.136 (0.691–1.867)	0.616
Microsatellite lesions	1.261 (0.740–2.147)	0.394		
BCLC stage B	1.264 (0.608–2.628)	0.530		
Child–Pugh B	2.918 (1.546–5.509)	0.001	2.633 (1.342–5.165)	0.005
Open surgery	2.281 (1.402–3.710)	0.001	1.918 (1.150–3.201)	0.013

*HCC* hepatocellular carcinoma, *MAFLD* metabolic dysfunction-associated fatty liver disease, *BMI* body mass index, *T2DM* type 2 diabetes mellitus, *MD* metabolic dysregulation, *AFP* alpha-fetoprotein, *BCLC* Barcelona Clinic Liver Cancer

^a^The diagnostic criteria for MAFLD include BMI and T2DM

Multivariate logistic regression analysis revealed that MAFLD independently increased the risk of major complications after radical resection in HCC patients (OR 1.859, 95% CI 1.106–3.124, *P* = 0.019). The other independent risk factors included: age ≥ 60 years, tumor diameter ≥ 5 cm, Child–Pugh grade B and open surgery (all *P* < 0.05) (Table 7).

### Subgroup analysis of HBV-HCC

HBV-HCC subgroup was analyzed due to the fact that 91.1% (853/936) HCC were diagnosed with HBV-HCC. The HBV-HCC patients were aged 57 years (49.0–64.0 years), including 698 males (81.8%) and 155 females (18.2%). The proportion of patients with BMI ≥ 23 kg/m^2^, T2DM, and MD were 51.1% (436), 14.1% (120), and 34.1% (291) respectively. They were divided into two groups based on the presence or absence of MAFLD: 178 (20.9%) patients in the MAFLD group and 675 (79.1%) patients in the non-MAFLD group. The baseline characteristics of patients in the HBV-HCC subgroup and the comparison of baseline characteristics between the MAFLD group and the non-MAFLD group were presented in Table 8.

**Table 8:** Baseline characteristics of patients with HBV-HCC subgroup

Variables	Patients (*n* = 853)	MAFLD (*n* = 178)	Non-MAFLD (*n* = 675)	*P* value
Age (years)	57.0 (49.0–64.0)	57.0 (48.0–62.0)	57.0 (49.0–64.0)	0.498
Male	698 (81.8%)	138 (77.5%)	559 (82.8%)	0.105
BMI (kg/m^2^)	22.9 (21.1–24.9)	24.2 (22.6–25.8)	22.4 (20.8–24.5)	< 0.001
BMI ≥ 23 (kg/m^2^)	436 (51.1%)	140 (78.7%)	296 (43.9%)	< 0.001
T2DM	120 (14.1%)	46 (25.8%)	74 (11.0%)	< 0.001
MD	291 (34.1%)	90 (50.6%)	201 (29.8%)	< 0.001
Excessive alcohol consumed	88 (10.3%)	24 (13.5%)	64 (9.5%)	0.118
HBV DNA (≥ 500 IU/mL)	762 (89.3%)	165 (92.7%)	597 (88.4%)	0.102
Cirrhosis	690 (80.9%)	151 (84.8%)	539 (79.9%)	0.133
Child–Pugh grade				0.140
A	793 (93.0%)	161 (90.4%)	632 (93.6%)	
B	60 (7.0%)	17 (9.6%)	43 (6.4%)	
Leukocyte count (× 10^9^/L)	5.5 (4.5–6.6)	5.5 (4.6–6.4)	5.5 (4.5–6.6)	0.989
Hemoglobin (g/L)	143.0 (138.0–152.0)	141.0 (136.0–153.3)	143.0 (139.0–152.0)	0.512
Platelet count (× 10^9^/L)	168.0 (148.5–206.5)	170.0 (147.5–198.0)	167.0 (150.0–208.0)	0.762
Prothrombin time (s)	13.3 (12.7–13.9)	13.3 (12.6–13.7)	13.4 (12.7–13.9)	0.124
Albumin (g/L)	40.0 (38.0–43.0)	40.0 (38.0–43.0)	40.0 (38.0–43.0)	0.923
Total bilirubin (µmol/L)	16.3 (12.0–21.8)	16.6 (11.7–22.8)	16.3 (12.0–21.6)	0.732
ALT (IU/L)	33.0 (23.0–49.5)	35.0 (27.0–51.3)	32.0 (23.0–49.0)	0.029
AFP (µg/L)	51.3 (6.3–697.4)	61.5 (6.2–222.8)	48.0 (6.4–843.0)	0.292
Tumor diameter (cm)	4.0 (2.7–6.7)	4.0 (3.0–6.1)	4.0 (2.5–7.0)	0.905
Number of tumors				0.569
1	744 (87.2%)	153 (86.0%)	591 (87.6%)	
≥ 2	109 (12.8%)	25 (14.0%)	84 (12.4%)	
Tumor cell differentiation				0.100
Well	12 (1.4%)	4 (2.2%)	8 (1.2%)	
Moderate	396 (46.4%)	71 (39.9%)	325 (48.1%)	
Poor	445 (52.2%)	103 (57.9%)	342 (50.7%)	
Tumor capsule				0.072
Complete	190 (22.3%)	46 (25.8%)	144 (21.3%)	
Incomplete	541 (63.4%)	100 (56.2%)	441 (65.3%)	
No tumor capsule	122 (14.3%)	32 (18.0%)	90 (13.3%)	
Microvascular invasion	468 (54.9%)	95 (53.4%)	373 (55.3%)	0.652
Microsatellite lesions	189 (22.2%)	35 (19.7%)	154 (22.8%)	0.368
BCLC stage				0.914
0	8 (0.9%)	2 (1.1%)	6 (0.9%)	
A	763 (89.4%)	160 (89.9%)	603 (89.3%)	
B	82 (9.6%)	16 (9.0%)	66 (9.8%)	
Surgical method				0.754
Open	445 (52.2%)	91 (51.1%)	354 (52.4%)	
Laparoscopic	408 (47.8%)	87 (48.9%)	321 (47.6%)	

*HBV-HCC* hepatitis B virus-related hepatocellular carcinoma, *MAFLD* metabolic dysfunction-associated fatty liver disease, *BMI* body mass index, *T2DM* type 2 diabetes mellitus, *MD* metabolic dysregulation, *ALT* alanine aminotransferase, *AFP* alpha-fetoprotein, *BCLC* Barcelona Clinic Liver Cancer

### Complications after radical resection in the subgroup of HBV-HCC patients

The overall morbidity rate of complications after radical resection in HBV-HCC patients was 20.9% (178/853). Classification of postoperative complications according to CCI: CCI (8.7–20.8) 44 (5.2%) patients, CCI (20.9–26.1) 63 (7.4%) patients, CCI (26.2–33.6) 21 (2.5%) patients, CCI (33.7–42.3) 15 (1.8%) patients, CCI (42.4–46.1) 15 (1.8%) patients, and CCI (46.2–100) 20 (2.3%) patients. Additionally, major complications (CCI ≥ 26.2) occurred in 69 (8.1%) patients (Tables 9 and 10).

**Table 9:** Incidence of postoperative complications in HBV-HCC patients

Complications	n (%)
Ascites	96 (11.3%)
Pleural effusion	92 (10.8%)
Intra-abdominal infection	72 (8.4%)
Pneumonia	67 (7.9%)
Liver failure	25 (2.9%)
Wound infection	16 (1.9%)
Intra-abdominal hemorrhage	13 (1.5%)
Hepatic encephalopathy	10 (1.2%)
Bile leakage	4 (0.5%)
Sepsis	4 (0.4%)
Acute renal failure	2 (0.2%)
Death	10 (1.2%)

*HBV-HCC* hepatitis B virus-related hepatocellular carcinoma

**Table 10:** CCI classification of postoperative complications in HBV-HCC patients

CCI	n (%)
0	675 (79.1%)
8.7–20.8	44 (5.2%)
20.9–26.1	65 (7.6%)
26.2–33.6	19 (2.2%)
33.7–42.3	15 (1.8%)
42.4–46.1	15 (1.8%)
46.2–100	20 (2.3%)
≥ 26.2	69 (8.1%)

*HBV-HCC* hepatitis B virus-related hepatocellular carcinoma, *CCI* comprehensive complication index

The incidence of postoperative complications in the MAFLD group was higher compared to the non-MAFLD group (*P* = 0.08). Moreover, the MAFLD group also exhibited a higher occurrence of infectious and major complications (CCI ≥ 26.2) compared to the non-MAFLD group (all *P* < 0.05) (Table 11).

**Table 11:** Comparison of complications between MAFLD group and non-MAFLD group

Variables	MAFLD (*n* = 178)	non-MAFLD (*n* = 675)	*P* value
Complications	50 (28.1%)	128 (19.0%)	0.008
Infectious complications	44 (24.7%)	92 (13.6%)	< 0.001
Major complications (CCI ≥ 26.2)	21 (11.8%)	48 (7.1%)	0.041

*MAFLD* metabolic dysfunction-associated fatty liver disease, *CCI* comprehensive complication index

In order to further elucidate the impact of different subtypes of MAFLD on post-hepatectomy complications in patients with HBV-HCC, they were divided into two groups based on their BMI: lean MAFLD group (BMI < 23 kg/m^2^) (38, 21.3%) and non-lean MAFLD group (BMI ≥ 23 kg/m^2^) (140, 78.7%). However, the incidence of complications, infectious and major complications did not show any statistically significant difference between these two groups (34.2% vs 26.4%, *P* = 0.344; 34.2% vs 22.1%, *P* = 0.126; 15.8% vs 10.7%, *P* = 0.390; respectively). According to the presence or absence of T2DM, the patients with MAFLD were divided into two groups: T2DM-MAFLD group (46, 25.8%) and non-T2DM-MAFLD group (132, 74.2%). The incidence of complications, infectious and major complications in the T2DM-MAFLD group was significantly higher compared to the non-T2DM-MAFLD group (52.2% vs 19.7%, *P* < 0.001; 47.8% vs 16.7%, *P* < 0.001; 26.1% vs 6.8%, *P* < 0.001; respectively). According to the presence or absence of MD, the patients with MAFLD were divided into two groups: MD-MAFLD group (90, 50.6%) and non-MD-MAFLD group (88, 49.4%). The incidence of complications, including infectious and major complications, appeared to be higher in the MD-MAFLD group compared to the non-MD-MAFLD group, however, these differences did not reach statistical significance (33.3% vs 22.7%, *P* = 0.115; 30.0% vs 19.3%, *P* = 0.099; 12.2% vs 11.4%, *P* = 0. 859; respectively).

### Influencing factors of complications after radical resection in the subgroup of HBV-HCC patients

**Fig. 2:**
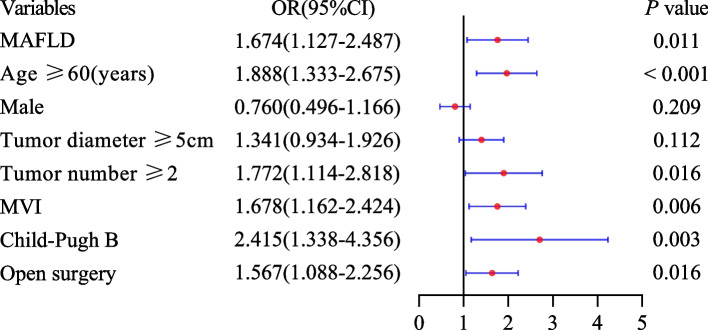
Influencing factors of complications after HBV-HCC hepatectomy

**Fig. 3:**
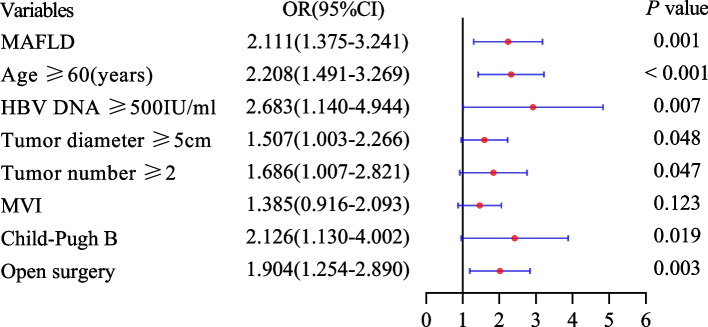
Influencing factors of infectious complications after HBV-HCC hepatectomy

**Fig. 4:**
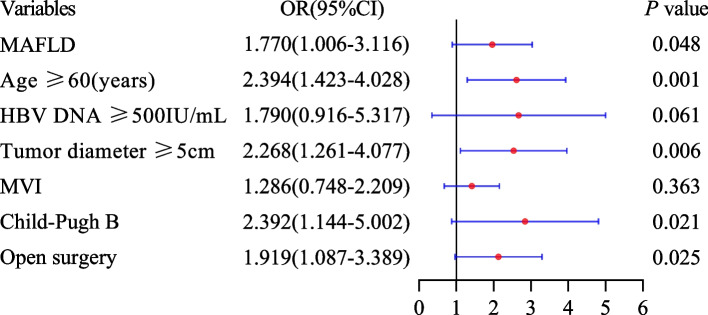
Influencing factors of major complications after HBV-HCC hepatectomy

Univariate logistic regression analysis found that MAFLD was a risk factor for complications after radical resection in HBV-HCC patients ((OR 1.669, 95%CI 1.142–2.439, *P* = 0.008). Multivariate logistic regression analysis showed that MAFLD was an independent risk factor for complications after radical resection in HBV-HCC patients (OR 1.674, 95%CI 1.127–2.487, *P* = 0.011) (Fig. 2). In addition, we also analyzed the influencing factors of infectious and major complications after radical resection in HBV-HCC patients. We also found that MAFLD was an independent risk factor for infectious and major complications after radical resection in HBV-HCC patients (OR 2.111, 95%CI 1.375–3.241, *P* = 0.001; OR 1.770, 95% CI 1.006–3.116, *P* = 0.048; respectively) (Figs. 3 and 4).

## Discussion

In this study, we retrospectively evaluated the impact of MAFLD on the complications after radical resection in HCC patients. The results revealed that MAFLD significantly increased the incidence of complications, including infectious and major complications after radical resection in HCC patients. Furthermore, MAFLD was identified as an independent risk factor for complications. Notably, the HBV-HCC patients with coexisting MAFLD and T2DM were particularly prone to developing postoperative complications.

With the escalating global prevalence of obesity and metabolic syndrome, the burden of MAFLD is rapidly increasing, particularly in the Asia–Pacific region [23]. The co-occurrence of HCC and MAFLD is increasingly prevalent due to the rising incidence of MAFLD. A considerable proportion of HCC patients were also found to have MAFLD in this study, specifically 21.5% (201/936) of HCC patients and 20.9% (178/853) of HBV-HCC patients. We also observed that the primary disparity in baseline characteristics were that MAFLD group exhibited a higher prevalence of metabolic disorders and elevated ALT levels compared to non-MAFLD group. However, the presence of MAFLD did not impact the pathological characteristics of patients with HCC. Similar findings were also noted in HBV-HCC patients. Previous studies [6, 15] have reported similar results, nevertheless, one of the studies found that patients within the MAFLD demonstrated better histological differentiation and lower rates of MVI compared to those without MAFLD, indicating earlier detection of HCC in patients with MAFLD. However, our study did not find any influence of MAFLD on histological differentiation and MVI. The reason may be that certain countries actively monitor MAFLD as a risk factor for HCC, leading to earlier detection of HCC in patients with concurrent MAFLD. In contrast, the recognition and surveillance of MAFLD in our country were still insufficient, resulting in no such disparity.

Therefore, the impact of MAFLD on the pathological characteristics of HCC requires further validation through multi-center and large-scale clinical as well as basic studies.

Hepatectomy has been extensively utilized for the treatment of various liver diseases. However, postoperative complication rates remain relatively high at approximately 20% to 56% [24]. This study found that the overall incidence of complications after radical resection in HCC and HBV-HCC patients were 21.0% and 20.9%, respectively. Therefore, the persistently high incidence of postoperative complications in patients with HCC is a challenging issue for surgeons in clinical practice [25]. Our study also found that the incidence of complications after radical resection in the MAFLD group was higher compared to the non-MAFLD group. Moreover, the presence of MAFLD independently contributed to an increased risk of postoperative complications in patients with HCC who undergo radical resection, suggesting that the coexistence of MAFLD was associated with an increased incidence of postoperative complications in patients with HCC.

This association can be attributed not only to the presence of hepatic steatosis in MAFLD patients but also to their higher susceptibility to metabolic disorders such as T2DM. Extensive evidence has consistently demonstrated that T2DM, as a metabolic disorder, significantly increases the incidence of complications following hepatectomy [26].

Considering that infectious complications is the most common post-hepatectomy complication in HCC patients, its incidence ranges from 4 to 25%, which is significantly associated with mortality risk [27, 28]. Therefore, it is crucial to identify and intervene in the risk factors associated with infectious complications following radical resection in order to effectively prevent infections and enhance the clinical outcomes of patients. In this study, a higher prevalence of post-hepatectomy infectious complications was observed among HCC and HBV-HCC patients, with rates of 15.6% and 15.9%, respectively. The present study employed the CCI to assess the severity of complications after radical resection in patients with HCC. It has been extensively utilized in assessing complications following abdominal surgery and is also widely referenced for evaluating complications after hepatectomy [29, 30]. The incidence of major complications (CCI ≥ 26.2) following radical resection in patients with HCC and HBV-HCC were relatively low (8.5% and 8.1%, respectively). We also found that MAFLD independently contributed to the risk of infectious and major complications after radical resection in HCC and HBV-HCC patients. The findings suggest that MAFLD may significantly increase the occurrence of infectious and major complications following radical resection in HCC patients.

In this study, we also observed that the HBV-HCC patients with T2DM-MAFLD group exhibited a higher occurrence rate of complications, including infectious and major complications compared to those with non-T2DM-MAFLD group. It is suggested that patients with HBV-HCC combined with T2DM-MAFLD are more susceptible to complications after radical resection. The reason for this is that hyperglycemia-induced oxidative stress response augmentation, inflammatory response enhancement, and impaired liver regeneration capacity [31]. Therefore, it is crucial to enhance the comprehension of MAFLD in patients undergoing radical resection for HCC and HBV-HCC, particularly MAFLD with T2DM. This will greatly contribute towards comprehensive preoperative evaluation and reduction in the incidence of postoperative complications.

Additionally, we also revealed that aged ≥ 60 years, Child–Pugh grade B, tumor diameter ≥ 5 cm, and open hepatectomy were identified as risk factors for post-hepatectomy complications, infectious and major complications in HCC and HBV-HCC patients, which is consistent with previous research findings [32–36].

This is because elderly patients may present with multiple comorbidities and experience gradual decline in organ function, resulting in compromised compensatory capacity of the liver and impaired regeneration ability of hepatocytes after radical resection [32]. Research has demonstrated that patients classified as Child–Pugh grade B (7 to 9 points) exhibit higher rates of postoperative complications and perioperative mortality compared to those Child–Pugh grade A (5 to 6 points) [33]. The prevailing belief both domestically and internationally is that the larger the diameter of a liver tumor, the broader the resection scope, and consequently, the more challenging the surgical procedure becomes with an increased likelihood of postoperative complications [34]. Compared to open surgery, laparoscopic surgery offers the advantages of reduced surgical trauma and faster postoperative recovery. A study of 3,876 HCC patients who underwent hepatectomy found that laparoscopic surgery was independently associated with lower incidences of postoperative infectious complications following hepatectomy for HCC compared with open surgery [35]. A meta-analysis also revealed that laparoscopic hepatectomy in HCC patients was significantly associated with decreased blood loss, successful R0 resection, wider scope of liver resection, shorter hospital stays, lower complication rates, and 30-day mortality [36]. Although BMI is an important criterion for diagnosing MAFLD, this study found no significant correlation between BMI and postoperative complications after HCC hepatectomy. Because the high BMI patients with HCC may have good nutritional and physiological reserves, leading to an enhanced inflammatory response to injury. This can potentially counteract postoperative complications in high BMI patients undergoing hepatectomy [37, 38].

There are inherent limitations to this study. Firstly, it is important to note that this study was conducted at a single center; however, the large sample size we collected helps mitigate potential selectivity bias to some extent. Secondly, our study population primarily consisted of HBV-HCC patients, accounting for over 90%. Further investigation is needed to determine the impact of MAFLD on complications after radical resection in HCC patients caused by different etiologies; however, this study demonstrates the detrimental effect of MAFLD on the complications after radical resection in HBV-HCC patients. Thirdly, it should be acknowledged that the present study is a retrospective analysis, wherein certain parameters such as waist circumference and HOMA-IR could not be extracted from electronic medical records, potentially resulting in a reduced diagnostic rate of MAFLD.

In conclusion, concurrent MAFLD was associated with a higher risk of complications, including infectious and major complications after radical resection in HCC patients, especially MAFLD with T2DM. It indicated that management of MAFLD may confer benefits in reducing complications after radical resection in HCC patients.

## Data Availability

No datasets were generated or analysed during the current study.
